# Diffracted X-ray Tracking Method for Measuring Intramolecular Dynamics of Membrane Proteins

**DOI:** 10.3390/ijms23042343

**Published:** 2022-02-20

**Authors:** Shoko Fujimura, Kazuhiro Mio, Tatsunari Ohkubo, Tatsuya Arai, Masahiro Kuramochi, Hiroshi Sekiguchi, Yuji C. Sasaki

**Affiliations:** 1 AIST-UTokyo Advanced Operando-Measurement Technology Open Innovation Laboratory (OPERANDO-OIL), National Institute of Advanced Industrial Science and Technology (AIST), 6-2-3 Kashiwanoha, Kashiwa 277-0882, Japan; shoko-san@aist.go.jp (S.F.); i180125b@yokohama-cu.ac.jp (T.O.); 2 Graduate School of Medical Life Science, Yokohama City University, 1-7-29 Suehiro-cho, Tsurumi-ku, Yokohama 230-0045, Japan; 3 Graduate School of Frontier Sciences, The University of Tokyo, 5-1-5 Kashiwanoha, Kashiwa 277-8561, Japan; t.arai@edu.k.u-tokyo.ac.jp (T.A.); masahiro.kuramochi.vw26@vc.ibaraki.ac.jp (M.K.); 4 Graduate School of Science and Engineering, Ibaraki University, Hitachi 316-8511, Japan; 5 Center for Synchrotron Radiation Research, Japan Synchrotron Radiation Research Institute, 1-1-1 Kouto, Sayo-cho, Sayo 679-5198, Japan; sekiguchi@spring8.or.jp

**Keywords:** single molecule analysis, conformation dynamics, membrane proteins, diffracted X-ray tracking technique

## Abstract

Membrane proteins change their conformations in response to chemical and physical stimuli and transmit extracellular signals inside cells. Several approaches have been developed for solving the structures of proteins. However, few techniques can monitor real-time protein dynamics. The diffracted X-ray tracking method (DXT) is an X-ray-based single-molecule technique that monitors the internal motion of biomolecules in an aqueous solution. DXT analyzes trajectories of Laue spots generated from the attached gold nanocrystals with a two-dimensional axis by tilting (*θ*) and twisting (*χ*). Furthermore, high-intensity X-rays from synchrotron radiation facilities enable measurements with microsecond-timescale and picometer-spatial-scale intramolecular information. The technique has been applied to various membrane proteins due to its superior spatiotemporal resolution. In this review, we introduce basic principles of DXT, reviewing its recent and extended applications to membrane proteins and living cells, respectively.

## 1. Introduction

Recent advances in cryo-electron microscopy have revealed structural information for various membrane proteins or supramolecular complexes at near atomic resolution that have been difficult to crystallize or inapplicable to NMR and in silico analysis, due to size limitation [1]. Simultaneously, there are growing expectations for knowledge on their structural dynamics. Every physiological event that we want to observe has a specific timeline to perform its function. The time resolution required for the measurement depends on the size of the observation target: whether observing the entire biological system, the cell behavior, or the internal dynamics of a single protein molecule. Fundamental processes on the molecular level, including bending, twisting, and rotation of the constitutive domains, occur on nanoseconds-to-seconds timescales [2,3]. Several single-molecule techniques have been applied to understand the dynamics of membrane proteins. These include fluorescence resonance energy transfer (FRET) [4,5], single-particle tracking (SPT) [6,7], single-molecule orientation tracking microscopy [8,9,10], atomic force microscopy/spectroscopy (AFM) [11], and diffracted X-ray tracking (DXT) [12,13,14,15,16,17]. However, real-time observation of structural rearrangements covering several timescales has proven to be more challenging.

In this review, we focus on DXT techniques for their high-resolution angular displacement and temporal resolution with microsecond-to-millisecond order. Accurate monitoring of the internal motion of protein molecules requires precision at the picometer level, which is three orders of magnitude lower than the nanometer level. This can be achieved not by visible light but with X-rays. The positioning accuracy of single molecule measurements is possible up to *λ*/100, where *λ* is the wavelength. Assuming that the wavelength ranges from 300 to 800 nm, single-molecule measurement technology using visible light has the positioning accuracy of several nanometers. If this is replaced by X-rays with a wavelength of 0.01–1 nm, a positioning accuracy can be achieved at the picometer level.

This imaging technique was established by Dr. Sasaki in 2000 to observe the movement of a single DNA molecule with picometer precision [18,19]. Since then, DXT has been applied to the dynamics analysis of various membrane proteins, such as bacteriorhodopsin [20]; a bacterial potassium channel, KcsA [12]; a nicotinic acetylcholine receptor (nAChR) [14]; transient receptor potential vanilloid 1 (TRPV1) [15]; metabotropic serotonin receptor subtype 2A (5-HT_2A_R) [16,17]; and molecular complexes, such as Group II chaperonin [13]. Additionally, DXT elucidates protein dynamics from a statistical analysis of thousands of trajectories generated from the movement of a single protein at a time. First, we introduce the basic principles of DXT, followed by key processing methods for data acquisition. Then, advances in data-analysis techniques to elucidate channel dynamics are discussed. Finally, we discuss the remaining challenges and further developments arising from the original DXT technique.

## 2. Principles of DXT

**Figure 1:**
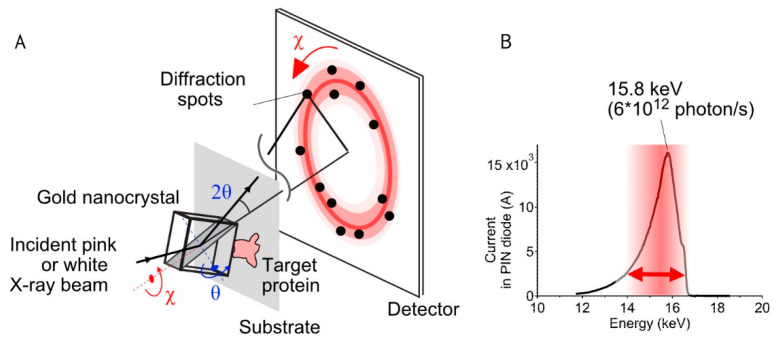
Schematic of the diffracted X-ray tracking method (DXT) principle. (**A**) Overview of the positions of the substrate, target proteins, Au nanocrystals, and detector. Since the X-ray beam has a wide energy band, the spots appear over a wide detector area according to Bragg’s law. The trajectories were analyzed on the *χ*-*θ* coordinates separately. (**B**) Current profile for the incident X-ray beam from the synchrotron radiation facility (BL40XU, SPring-8, Japan). The energy range of 14.0–16.5 keV (red arrow; undulator gap = 31 mm) was used for DXT measurements.

For DXT, specific domains of the target proteins were labeled by using gold nanocrystals, and the angular displacement of proteins was detected as a movement of the diffraction spots under a synchrotron radiation source with a high-speed detector. The orientation of the diffraction spots from the gold nanocrystals shifts accordingly to the structural change of the proteins (Figure 1A).

The motion of the diffraction spots from the gold nanocrystals follows Bragg’s law:

*nλ* = 2*d*sin*θ*

where *n* is the diffraction order, *λ* is the wavelength of the incident X-ray beam, *d* is the lattice plane spacing for a particular diffracting plane of atoms, and *θ* is the angle between the incident beam and the diffracting plane.

DXT uses broadband X-rays (a white or pink beam) instead of a monochromatic X-ray as the irradiation source. This enables us to track the angular displacement of the diffraction spots from individual gold nanocrystals (Figure 1B). Because the energy bands correspond to the wavelength width, the abovementioned equation can be interpreted as follows:

*n*Δ*λ* = 2*d*sinΔ*θ*

Assuming that the angle of the lattice plane is constant, the change in crystal orientation represents the change in the position of the diffraction spot on the detector at the XY plane within the range of Δ2*θ*. For *d* = 2.35 Å for Au(111), the twisting motion (Δ*θ*) of TRPV1 was obtained as 28.5 mrad from a pink beam of energy width ranging from 14.0 to 16.5 keV.

## 3. Framework to Link DXT Measurements with the Motion of Membrane Proteins

**Figure 2:**
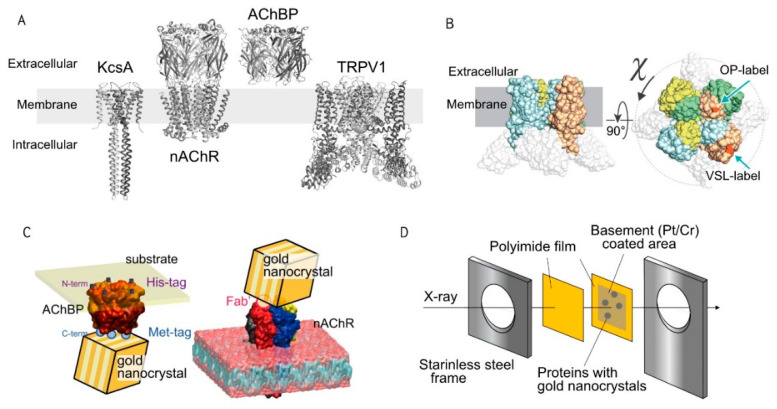
Design for diffracted X-ray tracking method (DXT) measurement. (**A**) Structures of KcsA (image was generated from PDB ID: 3EFF), nAChR (5KXI), AChBP (1I9B), and TRPV1 (3J5P). (**B**) Three-dimensional structure of TRPV1. The green arrows indicate positions of introduced Met-tags either at the outer pore (OP) or voltage-sensor-like (VSL) regions at the extracellular domain. The gold nanocrystals were specifically bound to the Met-tag (MGGMGGM) through Au-S binding. Reprinted with permission from American Chemical Society [15]. (**C**) Decoration of AChBP and nAChR with gold nanocrystals. The gold nanocrystal was immobilized to the AChBP C-terminus via a Met-tag (left) and to the extracellular side of the nAChR by the antibody F(ab’)_2_ fragment-gold nanocrystal conjugates (right) [14]. (**D**) Preparation of sample holder. After labeling the proteins with gold nanocrystals, they were covered with 5 µm–thick polyimide film with a 10 μL chamber buffer and sandwiched by a stainless-steel frame.

Membrane proteins play important roles in biological function, such as transmembrane trafficking, cell sensing and signaling, gas exchange, and energy synthesis. Almost 20–30% of genes encode membrane proteins in every organism. Chemicals and natural compounds that control the function of membrane proteins are actively being searched for as new drug candidates. Indeed, 50–60% of drugs on the market target membrane proteins. Ion channels are crucial components on the membrane surface that function as ion permeation through the hydrophobic lipid bilayer in response to various stimuli, such as chemical signals, physical signals, and changes in membrane potential. Ion channels undergo a series of intramolecular structural changes in response to stimuli and form an ion pass for ions to access the centrally located water-filled pore. The application of DXT has demonstrated the gating associated structural changes in KcsA, nAChR, and TRPV1 channels (Figure 2A).

## 4. Experimental Design

### 4.1. Labeling Proteins with Gold Nanocrystals

One important point of DXT experiments is to label selected protein domains with gold nanocrystals. Gold has many advantages to use as a probe in biophysical studies. It can bind directly and strongly to the thiol group of cysteine in protein molecules and increase the sensitivity of X-ray diffraction, due to its high density and mass number. Colloidal gold is one of the most widely used nanoparticles and easily available from companies in all sizes. However, colloidal golds are not suitable for DXT experiments, due to their low level of crystallization. Laue spots cannot be obtained from colloidal gold particles by using synchrotron radiation facilities. Therefore, to obtain sufficient diffractions, gold nanocrystals are generated by epitaxial growth on the surface of sodium chloride or potassium chloride crystals [21]. Gold is slowly vacuum deposited on the substrate surface of NaCl (100) or KCl (100) single crystals (0.001–0.01 Å/s) and grown epitaxially as a discontinuous film (island form). The substrate temperature is kept at about 375 °C while the gold crystals are growing. Then gold nanocrystals with a thickness of 1 nm and a diameter of 20–60 nm are achieved.

Since gold nanocrystals are fabricated in a vacuum, when they are stripped from the substrate for use in aqueous solution, aggregation starts immediately. Therefore, in order to change the hydrophobic nature of the nanocrystal surface to hydrophilic, a relatively high concentration of detergent was added to obtain a stable aqueous solution containing nanocrystals. It was confirmed that the detergent itself did not affect the efficacy of the subsequent reaction to the biomolecules. To obtain sufficient gold diffractions, gold nanocrystals with a diameter of 20–60 nm are used for DXT, the size of which is comparable to or larger than that of protein molecules.

The gold nanocrystals are introduced into the target proteins by gold–thiol bonds if the thiol groups are exposed on the protein surface [22]. Cysteine residues were introduced at the tip of each subunit of Group II chaperonin from *Thermococcus* strain KS-1 for Au binding, and their intramolecular motions were observed [13]. They used caged ATP as an ATP source to measure the rotational motion of the protein. Irradiation with UV light causes photolysis of the caged ATP and releases free ATP molecules. Prior to irradiation, the frequency of twisting motions was equally distributed between counterclockwise (CCW) and clockwise (CW) directions. After UV irradiation, the CW twisting movement disappears in 2 s and CCW rotation appears. This CCW motion relaxed after 5 s. This means that a CCW torsional motion is coordinated with the ring subunits, followed by a CW torsional motion to return to the original state representing a transition to the closed state.

Another technique to introduce Au binding site into the target protein is the site-specific introduction of the Met-tag (MGGMGGM), which takes advantage of the Au-S bond [23]. By introducing Met-tag, the capsaicin-induced twisting motion of TRPV1 was analyzed at positions near the ion pore (labeled at a loop between a helix 5 and a pore helix) and voltage-sensor-like domain (labeled at a loop between helices 1 and 2) independently [15] (Figure 2B).

Antibody-gold nanocrystal conjugates are frequently used to monitor the dynamics of target proteins. Cysteine residues in antibodies form sulfur bridges and strong attachments with the gold surface, so that the antibody and gold particles share electrons [24]. The dynamic motion of the extracellular side of nAChR was demonstrated by the gold-nanocrystal-conjugated F(ab’)_2_ fragments of the specific antibody [14] (Figure 2C).

Introducing commonly used peptide tags, such as FLAG-tag (DYKDDDDK), PA-tag (GVAMPGAEDDVV), and HA-tag (YPYDVPDYA), is also useful for the site-specific labeling and motion monitoring of proteins. Monoclonal antibodies against peptide tags are readily available from companies with lower prices, and their specificity is generally high. It is necessary to verify the conserved channel function in the mutant proteins. For calcium-permeable ion channels, we routinely applied cell-based calcium-permeable assay, using transfected cells.

### 4.2. Anchoring Target Proteins to the Polyimide Substrate

Another important point for preparing samples for DXT is developing a method for anchoring target proteins to the polyimide substrate. Kapton polyimide film (C_22_H_10_N_2_O_5_)_n_ was used as the substrate due to its excellent thermal stability and great resistance to chemicals and radiation (Figure 2D). The surface of the polyimide substrate was coated with chromium and gold (or platinum) by vapor deposition and used as a substrate surface for DXT. Because DXT elucidates protein dynamics by using the statistical analysis of thousands of trajectory data, target proteins are anchored to the substrate with aligned orientation at high density, and the flexibility of each segment must be retained.

Target proteins bearing a polyhistidine-tag can be immobilized to the substrate by using the Ni-NTA self-assembled monolayer. For TRPV1, a Met-tag was introduced into the extracellular loop to observe motion dynamics, and a (His)_6_ sequence was tagged at the N-terminus to be immobilized to the substrate (Figure 2B).

Target proteins containing primary amines (-NH_2_ groups) on the molecular surface can be directly anchored to the substrate by using the succinimidyl 3-(2-pyridyldithio) propionate (SPDP) reaction. Here, an amine-reactive N-hydroxysuccinimide ester reacts with lysine residues to form a stable amide bond. Thus, KcsA channel, which substituted cysteines with other amino acids beforehand, has other cysteines introduced to attach the gold nanocrystals [12]. The orientation of KcsA channels on the glass plate was fixed through a reaction between the specifically introduced lysine residues and SPDP. Other specific binding reactions, such as antigen–antibody reactions, biotin–avidin reactions, and glycoprotein–lectin reactions, can be used to anchor target proteins to the polyimide substrate.

## 5. Determination of Suitable Frame Rate and Data Processing

The frame rate of the detector determines the time resolution of DXT. Since the conformational dynamics of ion channels occur on nanoseconds to milliseconds timescales, high-speed recording system is required.

Previously, the DXT data of KcsA were recorded with a near video rate of 28 Hz, using the Hamamatsu photonics charge-coupled device camera C4880-82 in combination with an X-ray image intensifier (Hamamatsu Photonics). The data were collected at 18 °C because the diffraction spots were moving too fast to follow the movement across the frame over 20 °C [12]. The dynamics of KcsA are modulated by pH, and the motion along the *θ*-axis and *χ*-axis was predominant at a pH 7.5 and 4.0, respectively. Furthermore, DXT experiments further confirmed that the domain movement of KcsA was suppressed by the channel blocker tetrabutylammonium.

**Figure 3:**
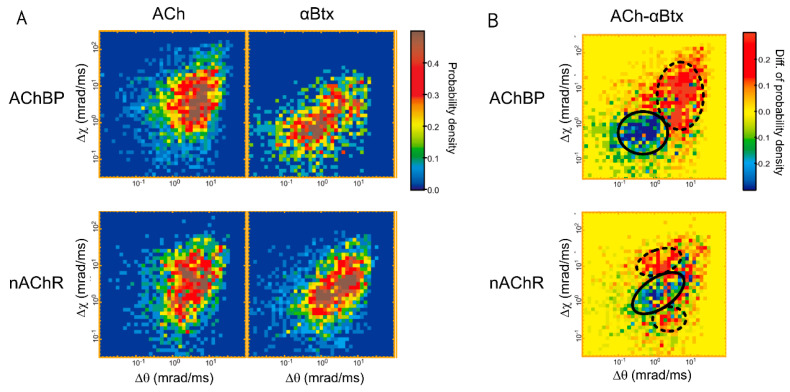
Two-dimensional histograms of DXT motion analysis of nAChR. (**A**) Two-dimensional histograms of AChBP and nAChR of the angular displacement over 100 μs time intervals. The horizontal and vertical axes are the tilting (*θ*) and twisting (*χ*) directions, respectively. Ach, acetylcholine; αBtx, α-bungarotoxin [14]. (**B**) Differential 2D histograms of AChBP and nAChR of the angular displacement over 100 μs time intervals. ACh and αBtx dominant areas are represented by dashed and solid circles, respectively [14].

A faster camera system FASTCAM SA1.1 (Photron Limited, Tokyo, Japan) combinated with an improved incident X-ray source and gold-nanocrystal quality enhanced the signal-to-noise ratio of data and enabled recording at a frame rate of 100 microseconds for AChBP and TRPV1 analysis [14,15,25]. AChBP is a homolog of the extracellular ligand-binding domain of nAChR and was used as a water-soluble model for the domain. This fast observation system detected local and intramolecular changes at room temperature. The detectable range of *θ* for AChBP and TRPV1 was 28.5 mrad. The 2D histograms of the angular displacement of the *χ*-axis and *θ*-axis for AChBP and nAChR were compared in the presence of acetylcholine (ACh) and α-bungarotoxin (αBtx) (Figure 3A). Differential 2D histograms showed that the Ach and αBtx modulate the dynamics: αBtx suppressed the ACh-induced motion in AChBP, while ACh generated multiple motion groups in nAChR (Figure 3B).

TRPV1 is a homotetrameric ion channel with a large size of 400 kDa and a complex structure containing more than twenty transmembrane segments. A structural comparison between the apo-(PDB:3J5P) and the fully open RTX/DkTx structure (PDB:3J5Q) suggests clockwise twisting at the extracellular domains associated with channel opening [26,27]. For the specific binding of gold nanocrystals, a Met-tag was introduced to a S1–S2 loop (for the “voltage-sensor-like-domain” label), or an S5-Pore loop (for the “pore-domain” label). Rotation angles of diffraction spots were separately analyzed at the positive (counterclockwise direction; CCW) and negative angles (clockwise direction; CW) viewed from the top (extracellular).

**Figure 4:**
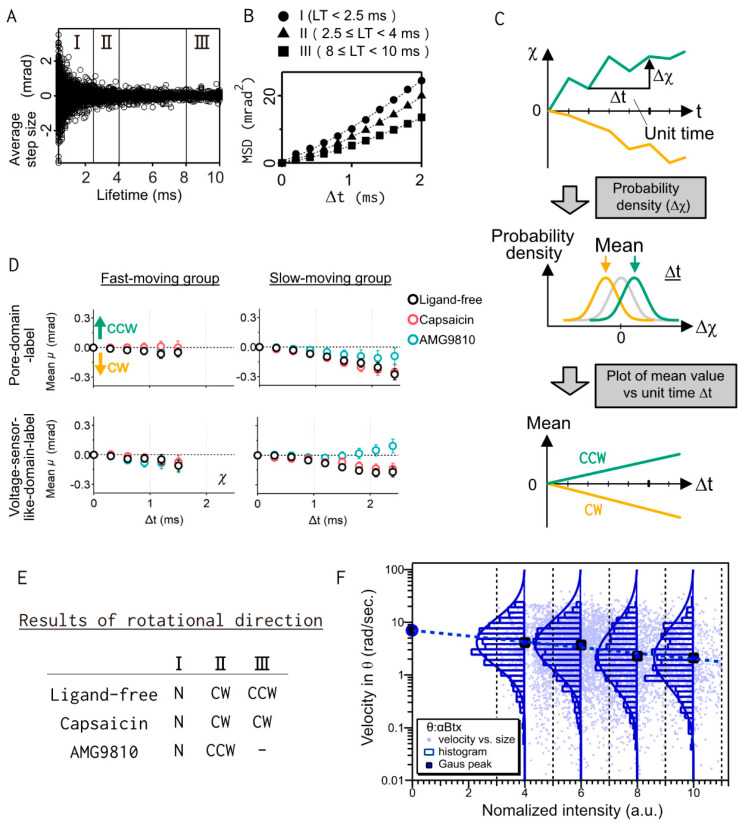
Data processing for the DXT analysis of TRPV1. (**A**) Lifetime filtering technique. The relationship between lifetime and angular displacement, *χ*. The results were categorized into three groups (Groups I–III) for analysis. (**B**) Mean-square displacement (MSD) for each lifetime group. (**C**) Data processing of TRPV1 dynamics. The probability densities were elucidated for each step size and were fitted with Gaussian curves. The Gaussian mean values were plotted against Δ*t*. The rotational bias can be determined as the slope of the mean plot. (**D**) Mean plots and 95% confidence intervals of the angle *χ*. The rotation angles of the fast-moving group were non-directed or slightly CW-biased (left panels). The mean plots at the slow-moving group showed ligand-dependent rotation bias (right panels). Reprinted with permission from American Chemical Society [15]. (**E**) Summary of rotational direction for each lifetime group. N, no bias; CW, clockwise rotation; CCW, counterclockwise rotation. (**F**) Relationship between the angular velocity in the *θ* direction and the normalized intensity of diffraction spots. The blue circle at the zero of the *x*-axis suggests the original motion without labeling [14].

To segregate the opening and closing motions, a lifetime filtering technique was applied [15]. Diffraction spots from gold nanocrystals can be recorded from the entire energy range of the incident of synchrotron radiation. The diffraction from fast-moving proteins have a short duration between appearance and disappearance, while those of slow-moving proteins have a longer duration. Therefore, the duration of the diffractions represents the overall movement (lifetime) of the target protein. The diffraction data were separated in three lifetime groups: Group I (lifetime (LT) < 2.5 ms), Group II (2.5 ≤ LT < 4 ms), and Group III (8 ≤ LT < 10 ms), and statistical analysis were applied independently (Figure 4A). This allowed us to detect ligand-induced internal motility of TRPV1. The diffusion constants, Dχ, obtained from the MSD curves were 9.87, 7.30, and 5.04 mrad^2^/ms, for groups I, II, and III, respectively, confirming that, the shorter the lifetime, the greater the motion (Figure 4B). The rotational bias of each group was calculated by using the probability density distribution of the step size. They were plotted as a mean value of the Gaussian fitting for Δ*t* (Figure 4C,D). In Group I, there was no rotation bias in the mean value plot, but in Group II, there was ligand-specific rotation bias. Negative bias was observed in the ligand-free and capsaicin condition, and positive bias was observed in the antagonist AMG9810 condition. In addition, there was a difference between ligand-free and capsaicin in Group III with longer lifetime (Figure 4E). From the cryo-EM structures of TRPV1, these results are thought to detect the transition from the closed to the open states of the channel upon capsaicin binding.

## 6. Live-Cell DXT: Measuring Protein Dynamics on Living Cells Using DXT

**Figure 5:**
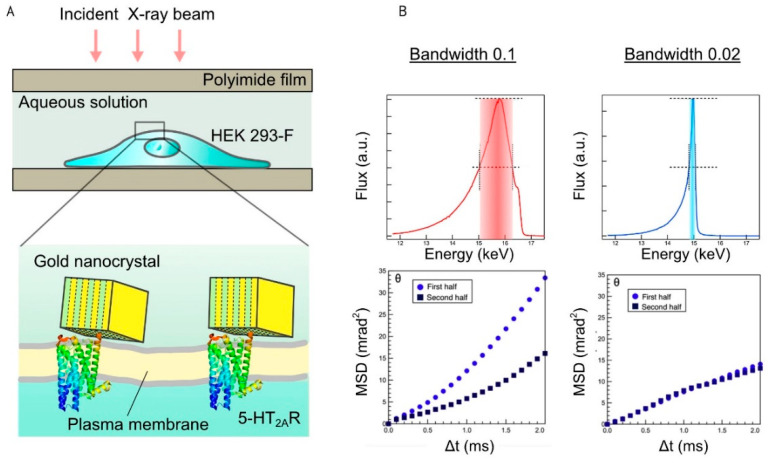
Live-cell diffracted X-ray tracking method (DXT) and X-ray damage assessment. (**A**) Experimental design for live-cell DXT measurement. The serotonin receptor 5-HT_2A_R was expressed on HEK293 cells, and then the N-terminally introduced FLAG-tag was labeled by using the FLAG antibody-conjugated gold nanocrystal. The cells were covered with 5 μm–thick polyimide films with 20 μL chamber buffer. (**B**) Assessment of cell damage in DXT. Left: Incident X-ray energy profile for bandwidth 0.1 (15.8 keV in peak energy and photon flux of 10^13^ photon/s) and the MSD curves obtained from 5-HT_2A_R motion analysis on live cells. Right: Incident X-ray energy profile for bandwidth 0.02 (15 keV in peak energy and photon flux of 10^13^ photon/s) and the MSD curves. Definition of bandwidth (Δ*E*/*E*); *E* is the maximum energy, and Δ*E* is full width at half maximum. The MSD curves of the bandwidth 0.1 showed a decreased angle in the second half, suggesting cell damage. The curves from the narrowed energy bandwidth of 0.02 showed no significant reduction in the second half. Reprinted with permission from Elsevier [16].

As an extended application of DXT for monitoring protein dynamics on living cells, live-cell DXT was developed. Cells and organelles contain biological membranes that shape them and support various biological activities. For the DXT experiments, membrane proteins were extracted from cells with detergent, and their motions were determined by DXT in the membrane protein–detergent complex. However, the membrane composition proved to be critical for gating of nAChR [28] and possibly other eukaryotic channels. Recently, we successfully applied the DXT technique to direct observation of the internal dynamics of 5-HT_2A_R in living cells (Figure 5A) [16]. The 5-HT_2A_R is a Gq-coupled GPCR, which activates phospholipase C. It was transiently expressed in HEK 293 cells, and the gold nanocrystals were attached to the N-terminally introduced FLAG-tag via anti-FLAG antibodies. The cells were then cultured in a CO_2_ incubator for 48 h to allow them to settle on the substrate surface. A lifetime filtering technique demonstrated that the unliganded receptors highly fluctuate with a clockwise twisting motion. However, this rotational motion was abolished by either a full agonist α-methylserotonin or an inverse agonist ketanserin.

Live-cell DXT is more sensitive to X-ray damage than normal protein DXT. X-ray damage includes direct and indirect damages. The indirect damage breaks chemical bonds or causes oxidation by free radicals due to the ionization of water [29]. To assess X-ray damage to the cells, the dataset was split in two (first half and second half), and their mean-square-displacement (MSD) curves were calculated individually (Figure 5B). The decrease in the slope of the second-half set indicates the effect of the X-ray damage. The shape of the MSD curve also shows a super-diffusion feature, suggesting that an external force, probably due to the thermal effect of X-rays, is applied. Examining various radiation conditions revealed that narrowing the X-ray bandwidth eliminates cell damage. After the measurement, cells were also checked by the microscope. There were no major changes in shape, nor the rupture of the cells themselves.

Another example of live-cell DXT is a dynamical analysis of the interleukin-2 receptor (IL-2R) and interleukin 15 receptor (IL-15R) on natural killer (NK) cells [30]. IL-15R is a transmembrane signaling protein consisting of three subsets: α, β (IL-15Rβ), and γ (γ_c_). IL-2 and IL-15 share the signaling domains IL-15Rβ and γ_c_, although they bind to intrinsic α-subsets and non-signaling domains. However, they have even contrasted physiological functions; indeed, they play different roles in activation-induced cell death (AICD). To understand why LI-2 and IL-15 generate contrasting signaling pathways, they applied live-cell DXT to NK cells and analyzed them from a molecular dynamics perspective. NK cells were cultured in the presence of nifuroxazide at the adherence step to induce inhibition. It was predicted that the dynamics of IL-2 and IL-15 would be more clearly discerned than those of IL-2/IL-15Rtrns and IL-15/IL-15Rtrns. The live-cell DXT showed that the motion of IL-15R was much reduced on the NK cells by IL-2 than by IL-15.

## 7. Diffracted X-ray Blinking Technique Using Monochromatic X-ray

Despite its excellent spatiotemporal resolution, the DXT method has not been widely used in the world so far. One of the limitations in using the DXT method is the use of white- or pink-beam X-rays for measurements. White- or pink-beam X-ray beamlines are not usually considered in the new beamline design, because more than 90% of users of conventional synchrotron radiation facilities use monochromatic X-rays. To make the DXT method available to a large number of users, a new DXT method utilizing monochromatic X-rays is needed.

The diffracted X-ray blinking (DXB) technique uses both low-dose monochromatic X-rays and nanocrystal labeling technology. It can observe internal molecular motion with wide timescales, ranging from microseconds to several seconds [30,31,32,33]. The intensity of X-ray diffraction from a moving single nanocrystal appears to blink during monochromatic X-ray irradiation because of Brownian motion in aqueous solutions. X-ray diffraction spots from moving nanocrystals were observed in the diffraction ring to cycle in and out according to Bragg’s law. If the protein molecule moves fast, the fluctuation of the diffraction spot rapidly shifts within the wavelength range. However, the diffraction spot fluctuates slowly if the protein motion is slow. Consequently, the internal motions of a protein molecule labeled with gold nanocrystals could be extracted from the statistical analysis of time-resolved trajectory data. This is a method used to characterize the movement of diffraction spots by measuring the autocorrelation function of X-ray diffraction intensities of each pixel and experimentally demonstrated that the method is surprisingly easy to use.

The time-resolved intensity fluctuation of each pixel around the Au(111) diffraction line, except for the intermodular rectangular area of the detector, was extracted and used for the analysis. The time course of the diffracted-photon signal, I(*t*), yielded information regarding the motions of the probe particles. The time-resolved intensity trajectory for each pixel was calculated by using the following autocorrelation function (ACF).

ACF = ⟨**I**(***t***)**I**(***t*** + ***τ***)⟩/⟨ **I**(***t***)^2^ ⟩,

where I(*t*) represents the diffracted photon intensity. The brackets, ⟨⟩, indicate time-averaged values. The calculated ACF was fitted to a single exponential curve by ACF(***τ***) = A · exp(−Τ ***τ***) + y, where A is the amplitude, y is the conversional value, Τ is the decay constant, and ***τ*** is the time interval. The parameters A and y were determined from the computed ACF data. The decay constant, Τ, was optimized to fit the ACF curve by using a nonlinear least-squares method. These calculations were performed for all pixels, and then the averaged ACF and distributions for the decay constants were generated from selected pixels by using the above conditions. This single-molecule diffraction measurement method using monochromatic X-rays cannot track all the movements of the resulting diffraction spots, such as DXT, but it can detect distinct blinking X-rays (X-ray blinking) by calculating the time-dependent autocorrelation function of the brightness intensity and elucidate the motion of a single protein molecule.

## 8. Future Perspectives

DXT successfully demonstrated the gating properties of three different ion channels: 1°/ms rotation for KcsA (maximum angle), 0.6°/ms for nAChR (normalized for the load from the gold nanocrystals), and 1.1–1.6°/ms for TRPV1 (maximum angle). DXT overcomes the problem of spatiotemporal resolution in the measurement of channel dynamics. Another experimental challenge is recording the structural dynamics and channel function simultaneously. Some successes have been reported, such as a combined measurement of FRET and single-channel patch-clamp [34,35], but few concurrent experiments were conducted due to the difficulty of the technology. This is a similarly challenging issue for DXT.

The size effect of the attached gold nanocrystals on protein dynamics must be considered in the DXT method. The relationship between the angular velocity in the *θ* direction of nAChR and the normalized intensity of the diffraction spots was analyzed (Figure 4F). Because the viscous resistances increased with respect to the size of the gold nanocrystals (calculated 3~4 folds from the size of used nanocrystals), the angular velocity of the larger gold nanocrystals was slightly decreased [14]. It was possible to determine the original motion without labeling with extrapolation of the *x*-axis to the zero.

The DXB method using monochromatic X-rays was developed to increase the number of DXT users who do not have easy access to white- or pink-beam X-ray sources. The DXB experiments can be performed by using a regular laboratory X-ray source device, as well as a large synchrotron radiation facility. It can be applied to living-cell motion analysis due to its reduced X-ray energy [16,30], and also to the measuring dynamics in material sciences, such as the microscale motions of silver halide crystals and crystalline polymers [32,33]. The DXB was first used for biological molecules by attaching gold nanocrystals. However, nanoparticle labeling was not required for crystalline materials, since their structure already results in an X-ray diffraction pattern. There is no doubt that the amount of information will be reduced, but the use of monochromatic X-ray has several advantages. The DXT method using a white- or pink-beam X-rays can track trajectories, but a signal-to-noise ratio in obtaining data is not very good, as it affects the detector system, reduces time resolution, and causes damage to the sample by using a higher X-ray flux than necessary. One of the advantages of monochromatic X-rays is the overwhelming reduction in X-ray flux, which clearly avoids the problem of X-ray damage in biomolecular systems. As mentioned above, the data reproducibility of monochromatic X-rays is much higher than the experimental results using white- or pink-beam X-rays even in the measurement using live cells. This is due to the reduction of damage to the sample. Time-resolved DXB measurements of a frame rate of 50 ms are now possible even with a laboratory X-ray source. This will enable the development of laboratory-scale instruments for X-ray single-molecule measurement. In the future, it may be possible to bring DXB devices to biosafety zones and analyze the dynamic behavior of the infectious materials, such as viruses. For this purpose, the miniaturization of DXB devices is awaited.

## Data Availability

Not applicable.
